# Compressed SENSE accelerated 3D single-breath-hold late gadolinium enhancement cardiovascular magnetic resonance with isotropic resolution: clinical evaluation

**DOI:** 10.3389/fcvm.2023.1305649

**Published:** 2023-11-30

**Authors:** Roman Johannes Gertz, Anton Wagner, Marcel Sokolowski, Simon Lennartz, Carsten Gietzen, Jan-Peter Grunz, Lukas Goertz, Kenan Kaya, Henrik ten Freyhaus, Thorsten Persigehl, Alexander Christian Bunck, Jonas Doerner, Claas Philip Naehle, David Maintz, Kilian Weiss, Christoph Katemann, Lenhard Pennig

**Affiliations:** ^1^ Institute for Diagnostic and Interventional Radiology, Faculty of Medicine and University Hospital Cologne, University of Cologne, Cologne, Germany; ^2^ Institute for Diagnostic and Interventional Radiology, Krankenhaus der Augustinerinnen, Cologne, Germany; ^3^ Department of Diagnostic and Interventional Radiology, University Hospital Würzburg, Würzburg, Germany; ^4^ Department III of Internal Medicine, Heart Center, Faculty of Medicine and University Hospital Cologne, University of Cologne, Cologne, Germany; ^5^ Kontraste Radiologie-Praxis Köln West, Cologne, Germany; ^6^ Radiologische Allianz Hamburg, Hamburg, Germany; ^7^ Philips GmbH, Hamburg, Germany

**Keywords:** cardiovascular magnetic resonance, late gadolinium enhancement, isotropic resolution, ischemic cardiomyopathy, non-ischemic cardiomyopathy

## Abstract

**Aim:**

The purpose of this study was to investigate the clinical application of Compressed SENSE accelerated single-breath-hold LGE with 3D isotropic resolution compared to conventional LGE imaging acquired in multiple breath-holds.

**Material & Methods:**

This was a retrospective, single-center study including 105 examinations of 101 patients (48.2 ± 16.8 years, 47 females). All patients underwent conventional breath-hold and 3D single-breath-hold (0.96 × 0.96 × 1.1 mm^3^ reconstructed voxel size, Compressed SENSE factor 6.5) LGE sequences at 1.5 T in clinical routine for the evaluation of ischemic or non-ischemic cardiomyopathies. Two radiologists independently evaluated the left ventricle (LV) for the presence of hyperenhancing lesions in each sequence, including localization and transmural extent, while assessing their scar edge sharpness (SES). Confidence of LGE assessment, image quality (IQ), and artifacts were also rated. The impact of LV ejection fraction (LVEF), heart rate, body mass index (BMI), and gender as possible confounders on IQ, artifacts, and confidence of LGE assessment was evaluated employing ordinal logistic regression analysis.

**Results:**

Using 3D single-breath-hold LGE readers detected more hyperenhancing lesions compared to conventional breath-hold LGE (*n* = 246 vs. *n* = 216 of 1,785 analyzed segments, 13.8% vs. 12.1%; *p* < 0.0001), pronounced at subendocardial, midmyocardial, and subepicardial localizations and for 1%–50% of transmural extent. SES was rated superior in 3D single-breath-hold LGE (4.1 ± 0.8 vs. 3.3 ± 0.8; *p* < 0.001). 3D single-breath-hold LGE yielded more artifacts (3.8 ± 1.0 vs. 4.0 ± 3.8; *p* = 0.002) whereas IQ (4.1 ± 1.0 vs. 4.2 ± 0.9; *p* = 0.122) and confidence of LGE assessment (4.3 ± 0.9 vs. 4.3 ± 0.8; *p* = 0.374) were comparable between both techniques. Female gender negatively influenced artifacts in 3D single-breath-hold LGE (*p* = 0.0028) while increased heart rate led to decreased IQ in conventional breath-hold LGE (*p* = 0.0029).

**Conclusions:**

In clinical routine, Compressed SENSE accelerated 3D single-breath-hold LGE yields image quality and confidence of LGE assessment comparable to conventional breath-hold LGE while providing improved delineation of smaller LGE lesions with superior scar edge sharpness. Given the fast acquisition of 3D single-breath-hold LGE, the technique holds potential to drastically reduce the examination time of CMR.

## 1. Introduction

Late gadolinium enhancement (LGE) cardiovascular magnetic resonance (CMR) is considered the gold standard for non-invasive myocardial tissue characterization (1, 2). Based on distinct enhancement patterns, LGE imaging enables the diagnosis of ischemic and different types of non-ischemic cardiomyopathies (1, 3–5). Additionally, the extent of LGE guides revascularization therapy in ischemic cardiomyopathy (6–8). It also carries prognostic value in both ischemic and non-ischemic cardiomyopathy by predicting the likelihood of adverse cardiovascular events (9–11).

Traditionally, most LGE sequences are acquired using inversion-recovery fast spoiled gradient-echo sequences in 2D multislice or 3D acquisition (2, 12, 13) during multiple predefined breath-holds. Nevertheless, these techniques hold limitations including anisotropic readouts with low through-plane resolution, signal-to-noise ratio (SNR) constraints, and the presence of partial volume effects (14–16). Previous works using conventional approaches employing longer breath-holds (15) or stack-of-spirals acquisition (17) have not allowed isotropic resolution to date.

To overcome these limitations, free-breathing LGE has been developed (18). This technique uses different approaches for respiratory synchronization and sequence types and thus provides a 3D high-resolution whole heart coverage (19–22). The acquired datasets, often yielding submillimeter isotropic resolution, can be reconstructed in any arbitrary view (18). The technique has demonstrated applicability in the depiction of left atrial fibrosis (23) and postablation scar (24) and facilitates an improved detection of smaller LGE lesions of the left ventricle (LV) (22, 25–28). However, despite the latest technical advances for acceleration of data acquisition and improving the efficiency regarding respiratory motion compensation, free-breathing LGE still suffers from long acquisition times from 3.5 to 12 min (29, 30). Hence, susceptibility to varying heart rate, respiratory motion, and changing contrast accumulation in the injured myocardium may lead to inadequate nulling of healthy tissue with subsequent limited feasibility and usefulness in clinical routine (31, 32).

In the past, Compressed SENSE, which combines parallel imaging using SENSitivity Encoding (SENSE) and compressed sensing (33, 34), was introduced. This acceleration technique enables an acquisition time which is lower than both types of acceleration techniques alone and has shown potential to accelerate the acquisition of imaging data in neurovascular (35, 36), cardiovascular (37, 38), and musculoskeletal imaging (39), especially for 3D isotropic datasets.

The purpose of this study was to investigate the clinical application of a Compressed SENSE accelerated single-breath-hold LGE sequence with 3D isotropic resolution by comparing assessment of LGE lesions and image quality to conventional LGE imaging acquired in multiple breath-holds.

## 2. Material & methods

### 2.1. Ethics

The institutional review board approved this single-center study (Ethikkommission, Medizinische Fakultät der Universität zu Köln, reference number: 21-1016). Given the retrospective design, the requirement for written informed consent was waived for the patient cohort.

### 2.2. Study population

The authors retrospectively reviewed the internal database at a tertiary care medical center for CMR studies performed between August 2020 and May 2021. Examinations were included if patients received a standardized protocol in clinical routine for assessment of ischemic or non-ischemic cardiomyopathies comprising both, 3D single-breath-hold LGE and conventional breath-hold LGE sequences at 1.5 T. Patients were excluded in case of (I) inadequate nulling of the LV myocardium, (II) strong breathing artifacts, and (III) severe arrhythmia.

The following data were recorded during examination or obtained from the medical charts: Patient age, body mass index (BMI) at examination date, gender, indication for CMR, left ventricular ejection fraction (LVEF) as assessed from short-axis (SA) cine images, heart rate during LGE imaging, and breath hold/acquisition time for LGE sequences. Final diagnosis of cardiomyopathies was reached in consensus by a board-certified cardiovascular radiologist and the treating cardiology consultant. Diagnosis was based on CMR and multidisciplinary evaluation of clinical data, laboratory examination, biopsy results if available, and echocardiographic findings.

### 2.3. CMR

CMR examinations were conducted in supine position using a commercially available whole body 1.5 T MRI system (Philips Ingenia, *Philips Healthcare, Best, The Netherlands*) with a dedicated 28-channel coil for cardiac imaging. The imaging protocol comprised 2D balanced steady-state free precession (bSSFP) cine sequences in standard orientations (SA, 4-chamber (4CH), 2-chamber (2CH), and 3-chamber (3CH)) during end-expiratory breath-hold followed by admission of Gadobutrol (Gadovist, *Bayer HealthCare Pharmaceuticals, Berlin, Germany*; 0.2 mmol/kg) being injected into an antecubital vein (flowrate of 2 ml/s). Depending on the indication for CMR, additional T1- (pre- and postcontrast) and T2-mapping sequences as well as T2-weighted fat-saturated black blood sequences were acquired in SA.

#### 2.3.1. Conventional breath-hold LGE

Ten minutes after application of contrast agent, a 2D gradient-echo T1-weighted Look-Locker sequence was used to determine inversion time (TI) adjusted to null LV myocardium. As clinical standard, an ECG-triggered (end-diastolic) 3D anisotropic Cartesian T1-weighted inversion-recovery fast spoiled gradient-echo sequence was used. 3D acquisitions were preferred over 2D since 3D acquisition enables a significant reduction of acquisition times and higher SNR efficiency compared to 2D (14, 40). Scans were acquired during end-expiratory breath-holds covering the LV in standard orientations [SA (two stacks), 4CH, 2CH, and 3CH]. Breath-hold time for SA (per stack) and 4CH were $\frac{60}{\mathrm{Heart}\;\mathrm{rate}}\times 18(s)$ and for 2CH and 3CH $\frac{60}{\mathrm{Heart}\;\mathrm{rate}}\times 20(s)$. Spectral presaturation with inversion recovery (SPIR) was employed to avoid high-intensity signals from epicardial fat. Of note, no dedicated acceleration of image acquisition (e.g., parallel imaging or compressed sensing) was employed, as demonstrated in previous studies introducing breath-hold 3D acquisition (14, 40, 41).

From here on and throughout this manuscript, this sequence is referred to as “conventional breath-hold LGE”.

#### 2.3.2. 3D single-breath-hold LGE

**Figure 1:**
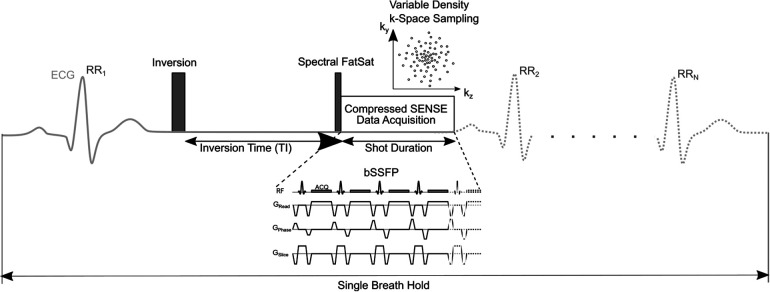
Schematic pulse sequence diagram of 3D single-breath-hold LGE. The sequence is (ECG-) triggered to end-diastolic cardiac phase with inversion time based on a previously acquired Look Locker sequence. Imaging data is acquired using balanced steady-state free precession (bSSFP) readout. In every cardiac cycle, 58 k-space lines were acquired leading to a shot length of 175 ms. Compressed SENSE was used for acceleration of data acquisition employing a variable density incoherent sampling patter with high-density in the k-space center and continuously decreasing sampling density towards the k-space periphery.

The employed sequence is an accelerated and modified version of a previously published free-breathing LGE sequence (22). Figure 1 shows a schematic pulse sequence diagram of the applied sequence.

After an additional Look-Locker sequence, 3D single-breath-hold LGE in transaxial plane was acquired immediately after conventional breath-hold LGE. To compensate for cardiac motion, ECG-triggering was employed. Right-left phase encoding direction was used to suppress potential breathing artifacts in patients with limited breath-hold capacity. In every cardiac cycle, 58 k-space lines were acquired leading to a shot length of 175 ms in end-diastolic cardiac phase. Like in conventional breath-hold LGE, SPIR was used for suppression of pericardial fat with bSSFP providing acquisition of data during end-expiratory breath-hold. bSSFP was used for data acquisition since it enables for a very short repetition time and high SNR efficiency, which is beneficial since less signal is lost due to signal spoiling compared to gradient-echo imaging and given the time constraints in high-resolution isotropic LGE. Breath-hold time of 3D isotropic single-breath-hold LGE was calculated as follows: $\frac{60}{\mathrm{Heart}\;\mathrm{rate}}\times 18(s)$.

Compressed SENSE (*Philips Healthcare, Best, The Netherlands*) with an acceleration factor of 6.5 was used for data acquisition. This technique utilizes a balanced variable density incoherent sampling pattern, with high-density in the k-space center and decreasing sampling density towards the k-space periphery. To mitigate motion artifacts, low-high k-space sampling was employed to collect data from k-space center first during mid-diastole and high frequency/resolution image information in the k-space periphery was sampled at the end of every shot. For reconstruction of imaging data, an iterative L1 norm minimization was used that ensured data consistency and sparsity in the wavelet domain being combined with regularization by coil sensitivity distribution and SENSE parallel imaging (33). Image reconstruction was performed employing standard hardware provided by the manufacturer of the MRI system and lasted about 15 s. Strong denoising algorithm was used for reconstruction of data as provided as a reconstruction setting for Compressed SENSE by the vendor. This algorithm adjusts the regularization of the wavelet denoising as employed in the Compressed SENSE reconstruction and counteracts the increase of image noise due to high Compressed SENSE factors.

From here on and throughout this manuscript, this sequence is referred to as “3D single-breath-hold LGE”.

Imaging parameters of LGE sequences are listed in Table 1.

**Table 1:** Comparison of scan parameters between conventional breath-hold LGE and 3D single-breath-hold LGE.

	Conventional breath-hold LGE	3D single-breath-hold LGE
Acquisition type	3D Cartesian	3D Cartesian
Sequence	T1w IR fast spoiled gradient-echo	bSSFP
ECG-triggering	End-diastolic	End-diastolic
Field of view, FH, LR, AP (mm^3^)	300 × 239 × 50 (4CH, SA)/280 × 204 × 50 (2CH, 3CH)	265 × 307 × 95
Matrix	172 × 119 (4CH, SA)/176 × 133 (2CH, 3CH)	120 × 140 × 86
Acquired resolution (mm^3^)	1.64 × 1.72 × 10	2.2 × 2.2 × 2.2
Reconstructed resolution (mm^3^)	1.59 × 1.59 × 10	0.96 × 0.96 × 1.1
Repetition time (ms)	3.5	1.42
Echo time (ms)	1.7	1.8
Flip angle (°)	15	45
Shot duration (ms)	170	175
Spectral fat suppression	Yes	Yes
Acceleration	Ø	Compressed SENSE 6.5, strong denoising

2CH, two-chamber; 3CH, three-chamber; 4CH, four-chamber; LGE, late gadolinium enhancement; SA, short-axis; bSSFP, balanced steady-state free precession; IR, inversion recovery.

### 2.4. Image analysis

For multiplanar reformatting of 3D single-breath-hold LGE source images, a radiologist with five years of experience in CMR (M.S.) created reconstructions in standard orientations (SA, 4CH, 2CH, and 3CH) with identical slice thickness as conventional breath-hold LGE scans. Reconstructions were created using the multiplanar reconstruction (MPR) tool in a commercially available image viewer (IMPAX EE, release 20; *Dedalus Healthcare Group, Bonn, Germany*).

In separate reading sessions using the same image viewer (IMPAX EE, release 20, *Dedalus Healthcare Group, Bonn, Germany*), the datasets were independently assessed by two radiologists with four (L.P., R1) and five (A.W., R2) years of experience in CMR and 3D isotropic LGE imaging. Both readers were blinded to clinical and patient data, respectively. First, readers evaluated conventional breath-hold LGE scans. After a period of four weeks to minimize recall bias, readers assessed 3D single-breath-hold LGE including its source images free to visualize in any arbitrary direction using the MPR tool and reconstructed standard orientations. Readers were allowed to adjust window leveling.

#### 2.4.1. Left ventricular LGE assessment

Readers evaluated scans for the presence of hyperenhancing lesions of the LV applying the American Heart Association's 17-segment model (42). Hyperenhancing lesions were scored for each segment based on the location (1 = subendocardial, 2 = mid-myocardial, 3 = subepicardial, and 4 = transmural) and the transmural extent (1 = 1%–25%, 2 = 26%–50%, 3 = 51%–75%, and 4 = 76%–100%). Additionally, scar edge sharpness of hyperenhancing lesions was assessed using a 5-point scale (1: non-diagnostic, 2: poor, 3: fair, 4: good, and 5: excellent). Further, readers analyzed scans for potential pericardial hyperenhancement (1 = left ventricular, 2 = right ventricular, and 3 circumferential) and intracardiac thrombi using a dichotomous yes/no question.

#### 2.4.2. Image quality, artifacts, and confidence of LGE assessment

Using 5-point scales, readers rated the image quality (IQ) (1: non-diagnostic, IQ unsuitable for diagnosis, 2: poor, suboptimal IQ for diagnosis, 3: fair, mediocre IQ sufficient for diagnosis, 4: good, image IQ confident diagnosis, and 5: excellent, IQ yielding highly confident diagnosis), artifacts including cardiac and respiratory motion artifacts, blurring artifacts, banding artifacts, and parallel imaging reconstruction artifacts (1: non-diagnostic, 2: high impact, 3: moderate impact, 4: low impact, and 5: none) as well as the confidence of LGE assessment (1: non-diagnostic, 2: low, 3: fair, 4: good, and 5: high) for both LGE techniques, respectively.

### 2.5. Statistical analysis

Data are expressed as mean ± standard deviation, unless noted otherwise. Normal distribution was tested using the Shapiro-Wilk test*.* Paired nonparametric variables as well as scan times were compared using Wilcoxon signed-rank tests. To compare the proportion of detected hyperenhancing lesions between LGE techniques, the McNemar test was applied. Cohen's Kappa was used to evaluate interobserver agreement of detection of hyperenhancing lesions using single-breath hold and conventional breath-hold LGE sequences. The interpretation of an agreement was as follows: 0.01–0.2 slight, 0.21–0.4 fair, 0.41–0.6 moderate, 0.61–0.8 substantial, and 0.81–0.99 almost perfect agreement (43).

Influence of confounders (heart rate, LVEF, BMI, and gender) on subjective evaluation of LGE sequences in terms of image quality, artifacts, and confidence of LGE assessment was assessed using ordinal logistic regression analysis. To exclude multicollinearity between variables, Pearsońs correlation coefficient was calculated.

Statistical analysis was performed using SPSS Statistics [Version 29.0.0.0 (241), IBM, Armonk, New York, USA]. Statistical significance was set to *p* < 0.05.

## 3. Results

### 3.1. Study population and baseline characteristics

One-hundred-and-twenty-three examinations met the inclusion criteria. Thereof, one examination was excluded given inadequate nulling in both techniques, three in conventional, and two in single-breath-hold LGE. Additionally, three examinations were excluded due to strong breathing artifacts in both, three in conventional, and four in 3D single-breath-hold LGE. Furthermore, two examinations were excluded due to severe arrythmia.

Consequently, 105 examinations of 101 patients (47 females) were included in this study, yielding an age of 48.2 ± 16.8 years (range: 17–78 years) and a BMI of 25.0 ± 4.3 (16.4–34.1). The mean LVEF was 62.7 ± 13.7% (13.0%–88.0%) and a heart rate of 69.5 ± 12.8 (38–102) beats per minute was observed during the examination. Scans were acquired for viability assessment in known/suspected non-ischemic cardiomyopathies in 81/105 (77.1%) and ischemic cardiomyopathy in 24/105 (22.9%) cases.

Indication for CMR and final diagnosis are listed in Table 2.

**Table 2:** Indications for CMR examinations and final diagnosis.

Indication for CMR (*n*, %)	105	Final diagnosis (*n*, %)	66
Non-ischemic cardiomyopathies	80 (76.2%)	Non-ischemic cardiomyopathies	51 (77.3%)
Amyloidosis	3 (2.9%)	Amyloidosis	1 (1.5%)
Anderson–Fabry disease	2 (1.9%)	Anderson–Fabry disease	1 (1.5%)
ARC	3 (2.9%)	ARC	1 (1.5%)
Carcinoid heart disease	1 (1.0%)	CCM	1 (1.5%)
Cardiac involvement in SLE	1 (1.0%)	Diffuse fibrosis	4 (6.1%)
Cardiac involvement in SSc	1 (1.0%)	Dilated cardiomyopathy	3 (4.5%)
CCM	2 (1.9%)	Hypertrophic cardiomyopathy	12 (18.2%)
Dilated cardiomyopathy	3 (2.9%)	Non-compaction cardiomyopathy	1 (1.5%)
Fibrosis in Tetralogy of Fallot	1 (1.0%)	Peripartum cardiomyopathy	1 (1.5%)
Hypertrophic cardiomyopathy	13 (12.4%)	(Peri-) Myocarditis	24 (36.4%)
Non-compaction cardiomyopathy	1 (1.0%)	Sarcoidosis	2 (3.0%)
Peripartum cardiomyopathy	1 (1.0%)		
(Peri-) Myocarditis	36 (34.3%)		
Sarcoidosis	1 (1.0%)		
Structural heart disease	8 (7.6)		
Takotsubo syndrome	3 (2.9%)		
Ischemic cardiomyopathy	23 (21.9%)	Ischemic cardiomyopathy	14 (21.2%)
Other	2 (1.9%)	Other	1 (1.5%)
Cardiac mass	2 (1.9%)	Cardiac mass	1 (1.5%)

Final diagnosis was based CMR, multidisciplinary evaluation of clinical data, laboratory examination, biopsy results if available, and echocardiographic findings. Thirty-nine patients did not show pathological findings in their examination. ARC, arrhythmogenic cardiomyopathy; CCM, chemotherapy-induced cardiomyopathy; SLE, systemic lupus erythematosus; SSc, systemic sclerosis.

### 3.2. Acquisition time

The mean acquisition of 3D single-breath-hold LGE was shorter than the combined acquisition time of conventional breath-hold LGE sequences (16 ± 3 s vs. 363 ± 87 s, *p* < 0.001).

### 3.4. Image analysis

#### 3.4.1. Left ventricular LGE assessment

**Figure 2:**
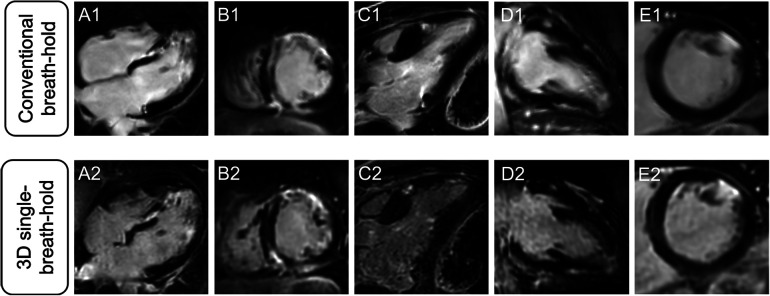
Comparison of different LGE patterns in conventional breath-hold LGE and 3D single-breath-hold LGE. Conventional breath-hold LGE (**A1–E1**) as reference standard and corresponding 3D single-breath-hold LGE reconstructions with the same slice thickness (10 mm) (**A2–E2**) in sarcoidosis (**A** and **B**), hypertrophic cardiomyopathy (**C**), myocarditis (**D**), and myocardial infarction (**E**).

Averaged segmental LGE assessment as well as individual evaluation of LGE are given in Table 3. Figure 2 displays exemplary comparisons of 3D single-breath-hold LGE and conventional breath-hold LGE in patients with ischemic and non-ischemic cardiomyopathies as proof of concept.

**Table 3:** Comparison of left ventricular LGE assessment between conventional breath-hold LGE and 3D single-breath-hold LGE with respect to localisation and transmural extend.

Averaged for both readers				
Overall detected hyperenhancing lesions in 1,785 segments				
Conventional breath-hold LGE	*n* = 216 detected hyperenhancing lesions (12.1%)			
3D single-breath-hold LGE	*n* = 246 detected hyperenhancing lesions (13.8%)			
Localization	***Subendocardial***	***Mid-myocardial***	***Subepicardial***	***Transmural***
Conventional breath-hold LGE	35.5/216 (16.4%)	42/216 (19.4%)	84/216 (38.9%)	54.5/216 (25.2%)
3D single-breath-hold LGE	43/246 (17.5%)	52/246 (21.1%)	95.5/246 (38.8%)	55.5/246 (22.6%)
Transmural extent	*1%–25%*	*26%–50%*	*51%–75%*	*76%–100%*
Conventional breath-hold LGE	47.5/216 (22.0%)	67.5/216 (31.3%)	46/216 (21.3%)	55/216 (25.5%)
3D single-breath-hold LGE	60.5/246 (24.6%)	79.5/246 (32.3%)	49.5/246 (20.1%)	56.5/246 (23.0%)
Reader 1				
Overall detected hyperenhancing lesions				
Conventional breath-hold LGE	*n* = 213 detected hyperenhancing lesions (11.9%)			
3D single-breath-hold LGE	*n* = 248 detected hyperenhancing lesions (13.9%)			
Localization	***Subendocardial***	***Mid-myocardial***	***Subepicardial***	***Transmural***
Conventional breath-hold LGE	36/213 (16.9%)	41/213 (19.2%)	84/213 (39.4%)	52/213 (24.4%)
3D single-breath-hold LGE	43/248 (17.3%)	53/248 (21.4%)	97/248 (39.1%)	55/248 (22.2%)
Transmural extent	*1%–25%*	*26%–50%*	*51%–75%*	*76%–100%*
Conventional breath-hold LGE	50/213 (23.5%)	66/213 (31.0%)	45/213 (21.1%)	52/213 (24.4%)
3D single-breath-hold LGE	65/248 (26.2%)	76/248 (30.7%)	51/248 (20.6%)	56/248 (22.6%)
Reader 2				
Overall detected hyperenhancing lesions				
Conventional breath-hold LGE	*n *= 219 detected hyperenhancing lesions (12.7%)			
3D single-breath-hold LGE	*n* = 244 detected hyperenhancing lesions (13.7%)			
Localization	***Subendocardial***	***Mid-myocardial***	***Subepicardial***	***Transmural***
Conventional breath-hold LGE	35/219 (16.0%)	43/219 (19.6%)	84/219 (38.4%)	57/219 (26.0%)
3D single-breath-hold LGE	43/244 (17.6%)	51/244 (20.9%)	94/244 (38.5%)	56/244 (23.0%)
Transmural extent	*1%–25%*	*26%–50%*	*51%–75%*	*76%–100%*
Conventional breath-hold LGE	45/219 (20.6%)	69/219 (31.5%)	47/219 (21.5%)	58/219 (26.5%)
3D single-breath-hold LGE	56/244 (23.0%)	83/244 (34.0%)	48/244 (19.7%)	57/244 (23.4%)

Averaged findings for both readers and for the respective readers are based on the American Heart Association's 17-segment model in terms of localization and transmural extent. Data are given as *n*/*n* (%). LGE, late gadolinium enhancement.

In 3D single-breath-hold LGE, readers detected more hyperenhancing lesions compared to conventional breath-hold LGE (*n* = 246 vs. *n* = 216 of 1,785 analyzed segments, 13.8% vs. 12.1%, *p* < 0.0001).

**Figure 3:**
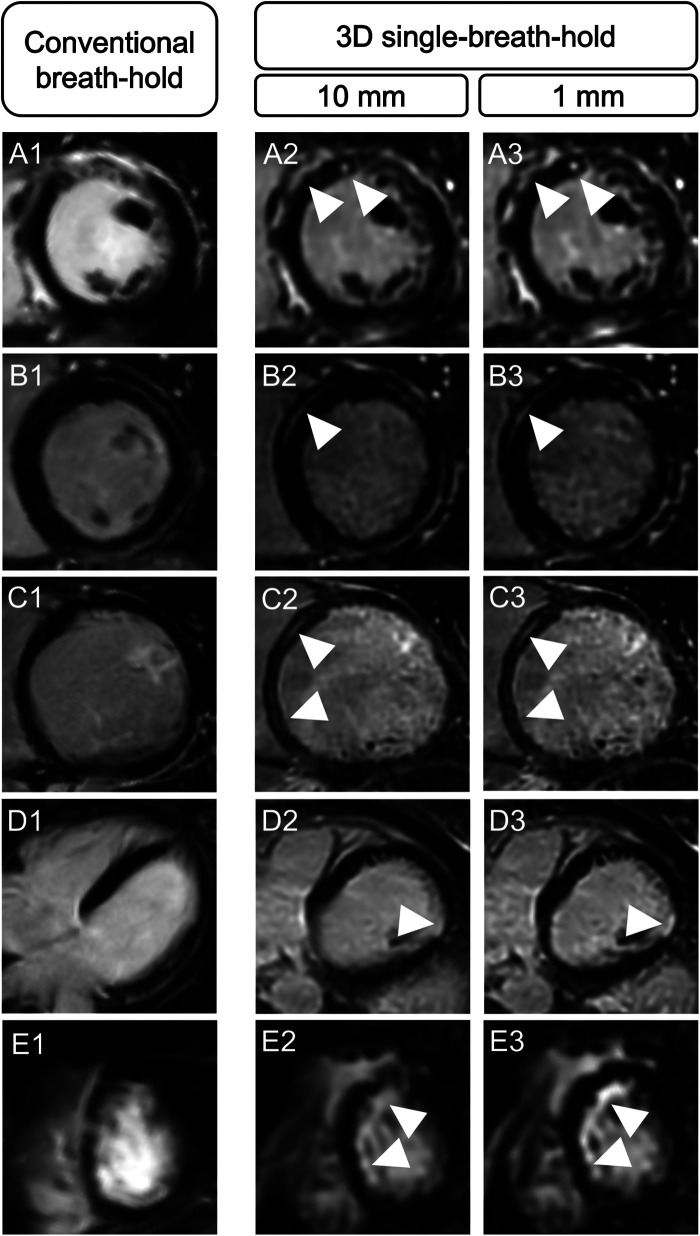
Illustration of the higher spatial resolution of 3D isotropic single-breath-hold LGE with additional LGE findings. Comparison of conventional breath-hold LGE and 3D isotropic single-breath-hold LGE reconstructions demonstrating additional LGE findings in 3D isotropic single-breath-hold LGE in patients with sarcoidosis (**A1–A3**, arrowheads), myocarditis (**B1–B3**, arrowheads), dilated cardiomyopathy (**C1–C3**, arrowheads), myocardial infarction with non-obstructive coronary arteries (**D1–D3**, arrowhead), and anterior wall infarction (**E1–E3**, arrowheads).

Averaged for both readers, depiction of transmural lesions was comparable between both techniques (3D single-breath-hold-LGE: 55.5, conventional breath-hold LGE: 54.5). In 3D single-breath-hold-LGE readers identified more lesions in subendocardial (43 vs. 35.5), mid-myocardial (52 vs. 42), and subepicardial localization (95.5 vs. 84). Additionally, readers were able to detect a higher number of small LGE lesions, as defined by a transmural extent <50%, using 3D single-breath-hold-LGE than with conventional breath-hold LGE (1%–25%: 60.5 vs. 47.5, 26%–50%: 79.5 vs. 67.5). Delineation of lesions with a larger transmural extent (>50%) was comparable between both techniques (3D single-breath-hold-LGE: 49.5, conventional breath-hold LGE: 46 for 51%–75%; 3D single-breath-hold-LGE: 56.5, conventional breath-hold LGE: 55 for 76%–100%). Figure 3 shows exemplary additional findings in 3D single-breath-hold-LGE in ischemic and non-ischemic cardiomyopathies.

Interobserver agreement for the detection of hyperenhancing lesions was excellent for both LGE techniques (3D single-breath-hold LGE: Cohen's Kappa of 0.93, conventional breath-hold LGE: Cohen's Kappa of 0.92).

**Figure 4:**
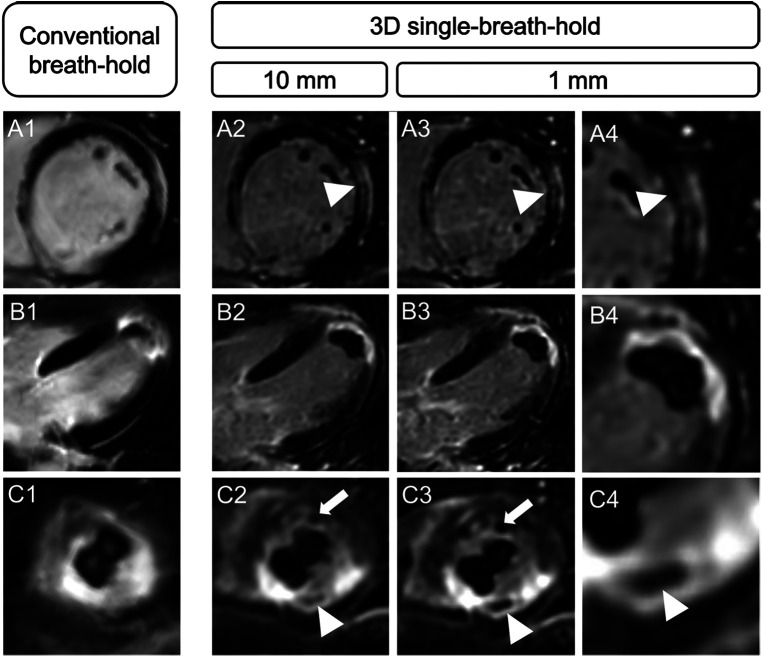
Comparison of spatial resolution and scar edge sharpness between conventional breath-hold LGE and 3D single-breath-hold LGE. Conventional breath-hold LGE and 3D isotropic single-breath-hold LGE reconstructions demonstrating the superior delineation of mid-myocardial LGE in sarcoidosis (**A**, arrowhead), subendocardial LGE in a patient with infarction of the left ventricular apex (**B**), the detection of microvascular obstruction (**C**, arrowhead; same patient as in **B**) and an additional left ventricular apex thrombus (**C**, arrow) in 3D isotropic single-breath-hold LGE.

Scar edge sharpness was rated as superior for 3D single-breath-hold LGE, by each individual reader and when averaging the results of both readers, respectively (R1: 4.2 vs. 3.4, R2: 3.9 vs. 3.2, averaged: 4.1 vs. 3.3; *p* for all <0.001) (Figure 4).

#### 3.4.2. Pericardial LGE and intracardiac thrombi

R1 identified pericardial LGE in 14 patients using both techniques with five patients presenting circular (35.7%) and nine with left ventricular (64.3%) hyperenhancement. R2 detected one more circular pericardial LGE using 3D single-breath-hold LGE than using conventional breath hold LGE [circular 7/15 (46.7%) vs. 6/14 (42.9%), left ventricular 8/15 (53.3%) vs. 8/14 (57.1%)].

**Figure 5:**
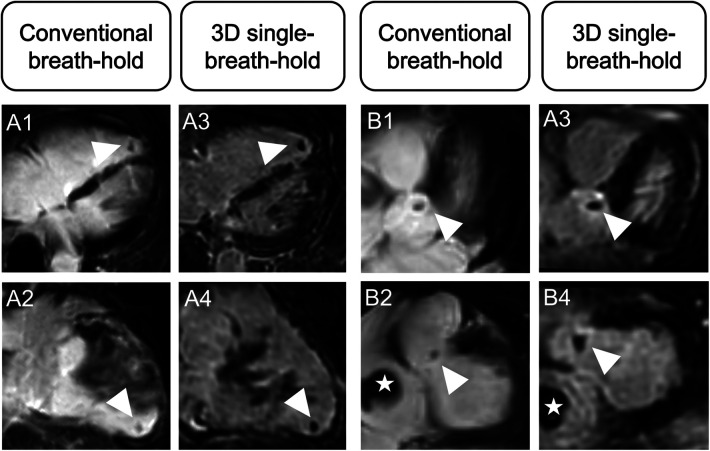
Advantage of higher spatial resolution and 3D multiparametric reconstructions of 3D single-breath-hold LGE. Conventional breath-hold LGE and 3D isotropic single-breath-hold LGE reconstructions demonstrating additional thrombus findings in 3D isotropic single-breath-hold LGE. (**A1–A4**) Right ventricular thrombus (arrowhead) in a patient with severe biventricular myocarditis, missed on initial scan. (**B1–B4**) Superior delineation of aortic valve associated mass (arrowhead) in a patient with right atrial thrombus (asterisk).

Both readers detected six intracardiac thrombi in five patients using 3D single-breath-hold LGE (*n* = 3 in the LV, *n* = 1 in the right ventricle, *n* = 1 in the right atrium, and *n* = 1 adjacent to the aortic valve). Using conventional breath-hold LGE, R1 found four (*n* = 2 in the LV, *n* = 1 in the right ventricle, and *n* = 1 in the right atrium) whereas R2 detected five (*n* = 2 in the LV, *n* = 1 in the right ventricle, *n* = 1 in the right atrium, and *n* = 1 adjacent to the aortic valve) thrombi in four patients (Figure 5).

#### 3.4.3. Image quality, artifacts, and confidence of LGE assessment

Based on the assessment of both readers, IQ (3D single-breath-hold LGE, 4.1 ± 1.0, conventional breath-hold LGE, 4.2 ± 0.9; *p* = 0.122) and diagnostic confidence (3D single-breath-hold LGE, 4.3 ± 0.9, conventional breath-hold LGE, 4.3 ± 0.8; *p* = 0.374) were comparable between both techniques. 3D single-breath-hold LGE was more severely affected by artifacts than conventional breath-hold LGE (3D single-breath-hold LGE, 3.8 ± 1.0, conventional breath-hold LGE, 4.0 ± 3.8; *p* = 0.002).

Considering the overall distribution of scores of both readers combined, Table 4 gives detailed results. Using conventional breath-hold LGE, readers graded scans of good/excellent image quality in 78.1%, low-impact/no artifacts in 72.8%, and with good/high diagnostic confidence in 83.8% of examinations. 3D single-breath-hold LGE provided scans with good/excellent image quality in 73.8%, with low-impact/no artifacts in 65.2%, and with good/high diagnostic confidence in 80.5% of cases.

**Table 4:** Comparison of image quality, artifacts, and diagnostic confidence between conventional breath-hold LGE and 3D single-breath-hold LGE.

Criterion	Technique	Non-diagnostic	Poor	Fair	Good	Excellent	Good + excellent
Image quality	Conventional	3 (1.4%)	5 (2.4%)	38 (18.1%)	70 (33.3%)	94 (44.8%)	164 (78.1%)
	Single	2 (1.0%)	13 (6.2%)	40 (19.0%)	66 (31.4%)	89 (42.4%)	155 (73.8%)
		Non-diagnostic	High impact	Moderate impact	Low impact	None	Low impact + none
Artifacts	Conventional	1 (0.5%)	10 (4.8%)	46 (21.9%)	75 (35.7%)	78 (37.1%)	153 (72.8%)
	Single	1 (0.5%)	20 (9.5%)	52 (24.8%)	79 (37.6%)	58 (27.6%)	137 (65.2%)
		Non-diagnostic	Low	Fair	Good	High	Good + high
Diagnostic confidence	Conventional	1 (0.5%)	6 (2.9%)	28 (13.3%)	67 (31.9%)	108 (51.4%)	175 (83.3%)
	Single	0 (0%)	10 (4.8%)	31 (14.8%)	64 (30.5%)	105 (50.0%)	169 (80.5%)

Data are given as *n* (%). Displayed scores are the averaged results for both readers. Conventional, conventional breath-hold LGE; LGE, late gadolinium enhancement; Single, 3D single-breath-hold LGE.

Table 5 displays the impact of possible confounders (LVEF, gender, heart rate, and BMI) on the evaluation of IQ, artifacts, and confidence of LGE assessment in both LGE sequences, as determined by ordinal logistic regression analysis. Female gender negatively influenced artifacts in 3D single-breath-hold LGE (*p* = 0.003) while increased heart rate led to decreased IQ in conventional breath-hold LGE (*p* = 0.003). LVEF and BMI did not yield a significant influence.

**Table 5:** Influence of confounders on subjective evaluation of LGE sequences: ordinal logistic regression analysis.

Criterion	Confounder	Conventional breath-hold LGE		3D single-breath-hold LGE	
		*p*	Beta	*p*	Beta
Image quality	Sex	0.345		0.355	
	BMI	0.229		0.979	
	Heart rate	**0** **.** **029**	**−0** **.** **215**	0.063	
	LVEF	0.122		0.066	
Artifacts	Sex	0.060		**0** **.** **028**	**0** **.** **232**
	BMI	0.391		0.914	
	Heart rate	0.225		0.098	
	LVEF	0.520		0.352	
Diagnostic confidence	Sex	0.417		0.157	
	BMI	0.634		0.814	
	Heart rate	0.111		0.098	
	LVEF	0.263		0.293	

Influence of heart rate, LVEF, BMI and gender on image quality, artifacts, and confidence of LGE assessment. Bold indicates statistical significance. BMI, body mass index; LGE, late gadolinium enhancement.

## 4. Discussion

In this study, we investigated the clinical application of Compressed SENSE accelerated 3D single-breath-hold LGE with isotropic resolution compared to conventional breath-hold LGE.

The major findings of the study are: (1) 3D single-breath-hold LGE enabled improved depiction of smaller hyperenhancing lesions of the LV in subendocardial, mid-myocardial, and subepicardial localization with superior scar edge sharpness. (2) 3D single-breath-hold LGE yielded image quality and confidence of LGE assessment comparable to conventional breath-hold LGE. (3) 3D single-breath-hold LGE required a drastically shorter scan time compared to conventional breath-hold LGE.

Given the limitations of conventional breath-hold LGE with anisotropic readouts, 3D isotropic LGE imaging techniques are gaining particular interest in both research and clinical practice (18). However, currently used free-breathing techniques for acquisition of 3D isotropic datasets suffer from long acquisition times with associated limitations hampering their application in clinical routine (18, 31, 32). Hence, the faster acquisition of 3D isotropic LGE datasets could pave the way for a broader usage in daily clinical practice. In this context, Gómez-Talavera et al. were the first to introduce an accelerated technique with a parallel imaging factor of 4. This allowed the acquisition of 3D LGE in a single breath-hold with an acquired resolution of 2.2 × 2.2 × 2.2 mm^3^ (44). However, the authors reported a long breath hold duration of 22 s, which likely restrains its application in patients with limited breath hold capacity (44).

In contrast, the proposed Compressed SENSE accelerated single-breath-hold technique facilitates the acquisition of 3D isotropic LGE with equal resolution as the aforementioned technique (44) during a breath hold duration of 16 s. Thus, the acquisition time is comparable to currently used conventional breath hold LGE techniques (44) and identical to SA and 4CH axes of the present study. The utilization of compressed sensing for accelerated data acquisition can result in considerable reconstruction time. This has been reported to last from one minute to about an hour for several free-breathing LGE techniques (27, 45–47) with some sequences requiring additional hardware employing central processing unit clusters (27, 45). On the contrary, the applied 3D single-breath-hold LGE technique does not require extensive reconstruction time. The image reconstruction process lasts about 15 s and can be performed with standard hardware provided by the manufacturer of the CMR system. Despite its short acquisition time, the proposed single-breath-hold LGE technique provides the acquisition of 3D isotropic datasets with a resolution (2.22 mm^3^ acquired and 0.96 × 0.96 × 1.1 mm^3^ reconstructed voxel size) comparable to several previously reported free-breathing LGE techniques with a longer acquisition time. In this regard, Bratis et al. reported a resolution of 2 mm^3^ acquired and 1 mm^3^ reconstructed voxel size in 4 min for an image-navigated technique (20), Bizino et al. of 1.7 mm^3^ acquired and 0.9 mm^3^ reconstructed voxel size in 09:30 min (28), and Andreu et al. of 2.7 mm^3^ acquired and 1.4 mm^3^ reconstructed voxel size in 16 min (48). Of note, recent studies have introduced free-breathing techniques that enable even higher resolutions beyond the proposed single-breath-hold LGE. In this respect, Akcakaya et al. (45) and Basha et al. (27) achieved a resolution ranging between 1 and 1.7 mm^3^ in 4–6 min, Rutz et al. reported a resolution of 1.15 mm^3^ in 8 min (49) and Zeilinger et al. attained a resolution of 1.3 mm^3^ in 11 min (47).

In line with previously reported studies comparing 3D isotropic LGE using free-breathing techniques to conventional breath-hold LGE (22, 25–28, 50), readers detected a higher amount of small hyperenhancing lesions of the LV using the single-breath-hold approach. This is due to its higher resolution in through-plane direction compared to conventional breath-hold LGE, which conversely suffers from partial volume effect due to its larger voxel size (16). These technical differences lead to an impaired detection of small hyperenhancing lesions in conventional breath-hold LGE, especially when these lesions are located directly adjacent to each other and are therefore challenging to distinguish as two separate findings. Additionally, the partial volume effect may also lead to inferior delineation of subepicardial and patchy mid-myocardial lesions. On the contrary, the higher resolution of 3D single-breath-hold LGE approach with possible 3D assessment enables differentiation from adjoining structures consequently leading to improved detection. Readers were able to detect more subendocardial lesions in 3D single-breath-hold LGE compared to conventional breath-hold LGE. This finding may be due to the higher resolution of 3D single-breath-hold LGE in through-plane direction and given the lower signal appearance of the blood pool in 3D single-breath-hold LGE since the data is acquired at the end of the examination, potentially allowing for an increased differentiation between myocardial lesions and the blood, comparable to dark-blood LGE (51–53). Of note, a quantitative assessment of IQ using SNR measurements was not conducted in this work given the iterative image and wavelet denoising reconstruction of the Compressed SENSE technique of 3D single-breath-hold LGE. This type of reconstruction is based on the actual image content which results in different numbers of denoising steps and iterations for different images leading to an interference with absolute estimations of the SNR.

In line with previous works comparing high-resolution LGE imaging with conventional LGE (28, 54), scar edge sharpness was rated superior in 3D single-breath-hold LGE. This is due to its higher through-plane resolution and consequently lower impact of partial volume effects, leading to a superior delineation of scars (16). Comparable to the published literature investigating free-breathing LGE (22), 3D single-breath-hold LGE provided a sufficient detection of pericardial hyperenhancement and intracardiac thrombi with slightly superior results compared to breath-hold LGE given its higher resolution (22). However, our findings pertaining to both intracardiac thrombi and pericardial LGE are limited given the low number of respective cases in this work.

3D single-breath-hold LGE yielded a slightly lower, but statistically not significant different IQ compared to conventional breath-hold LGE. Of note, the 3D single-breath-hold LGE was acquired at the end of the examination when patients tend to be exhausted and may have reduced breath-hold capacity. Whether an earlier acquisition of the 3D sequence might further increase its IQ is speculative based on our results and should thus be investigated in future studies.

The higher susceptibility to artifacts represents a limitation of 3D single-breath-hold LGE, mostly due to the limited breath-hold capacity of patients at the end of the examination leading to more pronounced artifacts. Nevertheless, this did not adversely affect diagnostic confidence, which was comparable between both techniques. The ability to visualize the 3D isotropic datasets in any arbitrary direction may counterbalance the detrimental effects of artifacts. These findings are comparable to previously reported studies comparing 3D isotropic LGE using free-breathing techniques and conventional breath-hold LGE (22, 50). Interestingly, female gender was associated with stronger artifacts in 3D single-breath-hold LGE. Given the right-left phase encoding direction, this is most likely due to hyperenhancing breast tissue, which leads to artifacts. Whereas increased heart rate led to decreased IQ in conventional breath-hold LGE, it did not negatively affect 3D single-breath-hold LGE, consistent to previously published work for 3D isotropic LGE using free-breathing (22).

The conventional breath-hold LGE technique in this study requires a total of 5 breath-holds for the respective axes. Considering the required recovery time between breath-holds along with potential repetition of scans due to non-compliant patients or inaccurate scan planning, this results in a long acquisition time of about 6 min. In contrast, 3D single-breath-hold LGE enables the acquisition of the entire LV in just one single breath hold, lasting about 16 s. This drastically reduces the overall scan time. Additionally, 3D single-breath-hold LGE proves to be widely user-independent since the sequence does not require adequate planning, as the respective axes can be reconstructed retrospectively in any desired orientation (18). This feature appears particularly appealing for radiographers with little experience in CMR-imaging.

Besides being a retrospective single-center investigation, there are several limitations to this study which need to be considered. First, given the distinct appearance of LGE techniques, a blinding of readers was not feasible possibly influencing the results. Second, the order of acquisition of LGE sequences was not randomized, potentially being disadvantageous for 3D single-breath-hold LGE. Third, 3D single-breath-hold LGE used bSSFP for data readout, which limits its direct comparison to conventional breath-hold LGE using spoiled gradient-echo. Fourth, the impact of the shot duration per cardiac cycle of 170 ms and 175 ms for the conventional breath-hold LGE and the 3D single-breath-hold LGE sequence, respectively, were not further investigated in the current work. Based on the observation in other studies it can be assumed, that shorter shot durations are of benefit especially for patients with high heart rates to effectively avoid motion artefacts or blurring due to cardiac motion in the images (17, 19, 20, 46, 47). However, reducing the shot duration per cardiac cycle directly increases total scan time and breath-hold length and would be needed to be offset by higher acceleration factors. Fifth, there was no reference standard for the detection of LGE lesions. Repeated examining of the patients on the following day or histopathological correlation could have validated additional findings of 3D isotropic LGE. However, neither was this possible nor desired given the study design and potential complications of endomyocardial biopsy (55). Sixth, the number of cases including intracardiac thrombi and pericardial enhancement were relatively low and did not allow for a sufficient analysis of potential benefits of 3D single-breath-hold LGE. Seventh, we did not assess the diagnostic accuracy of 3D single-breath-hold LGE for the detection of atrial fibrosis and right ventricular LGE. Given the thin myocardium of these chambers the higher spatial resolution of 3D single-breath-hold LGE may prove especially beneficial. Thus, assessing the techniques efficacy in these indications warrants further studies. Lastly, the chosen acceleration factor for 3D single-breath-hold LGE was based on our clinical experience with isotropic LGE imaging and not after dedicated evaluation of different undersampling factors. Hence, higher acceleration factors, preferably with deep learning-based image reconstructions (39) could further increase the resolution and decrease shot duration in each cardiac cycle and/or breath-hold duration, meriting further investigation in future research.

## 5. Conclusions

Compressed SENSE accelerated 3D single-breath-hold LGE provides a superior depiction of small hyperenhanced lesions of the LV in ischemic and non-ischemic localizations with higher scar edge sharpness while yielding comparable IQ and diagnostic confidence to conventional breath-hold LGE. Given the drastically reduced scan time of LGE imaging without extensive reconstruction time or required additional hardware, the proposed sequence can greatly expand the indication for CMR, improve patient management and care while reducing costs.

## Data Availability

The datasets generated and/or analyzed during the current study are not publicly available due to data protection but are available from the corresponding author upon reasonable request.
